# Supplementary material for: Radioimmunotherapy of methicillin-resistant Staphylococcus aureus in planktonic state and biofilms
Source: PLoS One. 2020 May 14;15(5):e0233086. doi: 10.1371/journal.pone.0233086 (PMC7224548; doi:10.1371/journal.pone.0233086)
Supplement: Attachment — Submitted filename: Response to the Reviewers.docx [file pone.0233086.s002.docx]

February 3, 2020

Dr. Amal Al-Bakri
Academic Editor
PLOS ONE

**Re: revision of the manuscript PONE-D-19-33702 Radioimmunotherapy of Methicillin-resistant Staphylococcus aureus in planktonic state and biofilms**

Dear Dr. Al-Bakri,

Thank you very much for giving us the opportunity to submit a revised manuscript. We have modified the manuscript according the Reviewers’ and Editorial Comments. Please find below the point by point responses to those comments.

We hope that you will find a revised manuscript suitable for publication in PLOS ONE.

Updated Funding Statement: This research was funded by Health Holland, which is non for profit organization, financed by the Netherlands Organization for Scientific Research (NWO), Grant number LSHM-17026. The funders had no role in study design, data collection and analysis, decision to publish, or preparation of the manuscript

Updated Competing Interests statement: The authors declare no conflict of interest.

Sincerely,

Ekaterina Dadachova, PhD

Reviewer 1

Though the manuscript is presented as a proof of concept, we recommend that the potential toxicity of such treatment to be highlighted in the manuscript. Accordingly, you are advised to modify the manuscript highlighting this limitation and resubmit.

**Response**: We have added the following paragraph and additional references 12-15 to the revised manuscript. The paragraph reads: “Previously we have performed extensive evaluation of RIT toxicity in mouse models of fungal and bacterial infections using the antibodies labeled with the same ^213^Bi radionuclide utilized in this work. No systemic toxicity was noted in mice infected with *Streptococcus pnemoniae* and treated with ^213^Bi-labeled antibody to bacterial polysaccharide (12). The toxicity evaluation of RIT-treated mice infected intratracheally with *C. neoformans* showed the absence of acute hematologic and long-term pulmonary toxicity (13). RIT was much better tolerated by the treated mice than Amphoterecin B which is a current standard of care for invasive fungal infections (14). In addition, RIT did not adversely affect bystander mammalian cells such as CHO cells and macrophages, with the latter being able to carry out their functions such as nitric oxide production after RIT exposure (15). As all these observations have been made for systemic administration of the radiolabeled antibodies, the anticipated local application of RIT into the infected joint should be even safer in this regard.”

Journal Requirements

When submitting your revision, we need you to address these additional requirements.

1. Please ensure that your manuscript meets PLOS ONE's style requirements, including those for file naming. The PLOS ONE style templates can be found at

http://www.journals.plos.org/plosone/s/file?id=wjVg/PLOSOne_formatting_sample_main_body.pdf and http://www.journals.plos.org/plosone/s/file?id=ba62/PLOSOne_formatting_sample_title_authors_affiliations.pdf

**Response**: We have modified the manuscript according to PLOS ONE style.

2. In your Methods section, please give the sources of any bacterial strains used in your study.

**Response**: We have included the source of bacteria into the Methods

3. We note that you have indicated that data from this study are available upon request. PLOS only allows data to be available upon request if there are legal or ethical restrictions on sharing data publicly. We will update your Data Availability statement on your behalf to reflect the information you provide. – **Response**: We have uploaded the data as a Supporting information file.

4. PLOS requires an ORCID iD for the corresponding author in Editorial Manager on papers submitted after December 6th, 2016. Please ensure that you have an ORCID iD and that it is validated in Editorial Manager. To do this, go to ‘Update my Information’ (in the upper left-hand corner of the main menu), and click on the Fetch/Validate link next to the ORCID field. This will take you to the ORCID site and allow you to create a new iD or authenticate a pre-existing iD in Editorial Manager. Please see the following video for instructions on linking an ORCID iD to your Editorial Manager account: <https://www.youtube.com/watch?v=_xcclfuvtxQ> – **Response**:

ORCID iD has been linked to the manuscript.

5. We note that you have included the phrase “data not shown” in your manuscript. Unfortunately, this does not meet our data sharing requirements. PLOS does not permit references to inaccessible data. We require that authors provide all relevant data within the paper, Supporting Information files, or in an acceptable, public repository. Please add a citation to support this phrase or upload the data that corresponds with these findings to a stable repository (such as Figshare or Dryad) and provide and URLs, DOIs, or accession numbers that may be used to access these data. Or, if the data are not a core part of the research being presented in your study, we ask that you remove the phrase that refers to these data. – **Response:** We are now showing this data in the modified figures.
