# Supplementary material for: Radioimmunotherapy of methicillin-resistant Staphylococcus aureus in planktonic state and biofilms
Source: PLoS One. 2020 May 14;15(5):e0233086. doi: 10.1371/journal.pone.0233086 (PMC7224548; doi:10.1371/journal.pone.0233086)
Supplement: Attachment — Submitted filename: Response to the Reviewers.docx [file pone.0233086.s003.docx]

**Second revision of the manuscript PONE-D-19-33702 Radioimmunotherapy of Methicillin-resistant Staphylococcus aureus in planktonic state and biofilms**

**Response to the Reviewers**

Reviewer #1:

*This manuscript expands on the growing repertoire of immunotherapy applications, outside of cancer. The authors have provided a proof-of-concept study investigating radioimmunotherapy for the treatment of Methicillin-resistant staphylococcus aureus. As such, rigorous experimentation as been performed and the data provided is sufficient. This research indeed contributes to a gap in the field of therapeutic options for prosthetic joint infection (PJI) and offers a novel approach. No additional revision is required, as authors have adequately addressed the comments of previous reviewer. Furthermore, this manuscript adheres to PLOS ONE criteria.* – **Response**: We would like to thank the Reviewer for his/her encouraging opinion about our work.

Reviewer #2:

*The authors have addressed the issues raised in the previous review. However, there is little innovation here. It is not clear that there is a specific antibody effect and whatever was observed would be expected. An in vivo study would generate more interest*. – **Response**: We would like to thank the Reviewer for acknowledging that we have addressed the issues in the previous review.

We respectfully disagree about the lack of the innovation. In the revised manuscript we have expanded the sentence in the Discussion stating that: “To the best of our knowledge, this is the first report of RIT treatment of MRSA in particular and of any multidrug resistant bacteria in general in both planktonic and biofilm state”.

We have also added the explanation about some non-specfic killing to the Discussion: “In this regard, the non-specific killing by both radiolabeled bacterium-specific and non-specific

antibodies results from one or combination of two events: 1) in vitro there is always some non-specific killing of cells by highly destructive alpha and beta particles emitted by the radionuclides in a small volume of an assay; 2) WTA-specific 4497 and RSV mAbs are human monoclonal antibodies which, on average, have much higher isoelectric points (IP) than murine antibodies. Antibodies with higher IPs have a tendency to non-specifically bind to the cells, therefore, both radiolabeled 4497 and RSV mAbs demonstrated some non-specific therapeutic effect towards bacterial cells. However, the killing effect of radiolabeled with ^213^Bi 4497 mAb was higher than that of radiolabeled RSV mAb under examined conditions, indicating that killing of MRSA by ^213^Bi-4497 mAb was WTA-specific”.

In regard to the animal experiments, they are out of scope of this proof of principle manuscript.

Currently on-going work in animal models on non-invasive nuclear imaging, therapy and safety evaluation will be reported in a follow-up manuscript. We have added a sentence to the conclusions that “the work in the animal models of MRSA infection is currently on-going”.
